# Lysozyme Activity in the Plasma of Rodents Infected With Their Homologous Trypanosomes

**Published:** 2012

**Authors:** S Maraghi, DH Molyneux, KR Wallbanks

**Affiliations:** 1 Department of Parasitology and Mycology, Abadan Arvand International Division, Infectious and Tropical Diseases, Thalassemia and Haemoglobinopathy Research Centers. Jundishapur University of Medical Sciences, Ahwaz, Iran; 2 Liverpool School of Tropical Medicine, Pembrooke Place, Liverpool L3, 5 QA UK

**Keywords:** Lysozyme, Rodents, *Trypanosoma*, *Herpetosoma*

## Abstract

**Background:**

In this study the concentration of lysozyme in blood plasma of *Microtus agrestis, Clethrinomys glareolus*, *Apodemus sylvaticus*, BK rats and outbred white mice before and after infection with culture forms of *Trypanosoma microti, T, evotomys, T. grosi, T. lewisi and T. musculi* respectively was measured.

**Methods:**

Blood samples of rodents, *Microtus agrestis, Clethrionomys glareolus, Apodemus sylvaticus*, BK rats and outbred mice infected with *T*. *microti, T. evotomys, T. grosi, T. lewisi* and *T. musculi* respectively were collected in heparinized micro- tubes immediately before inoculation and 3, 6, 12, 24, 48, 96 and more than 400 days after intra- perituneal inoculation with 5×10^5^of their homologous trypanosome parasites of which more than half were metacyclic trypomastigote in 0.2 ml of culture medium. Micro- tubes were centrifuged and plasma samples were separated and the lysozyme activity was measured by the agar method.

**Results:**

Levels of lysozyme rose rapidly three to six days after the inoculation to ten to twenty than their pre- infection levels. They then gradually decreased, although after more than one year they were still two to ten folds higher than controls. The highest level measured occurred in rats infected with *T. lewisi* and the lowest in *A. sylvaticus* infected with *T. grosi*. After one year the highest concentration of lysozyme was in mice infected with *T. musculi* and lowest in *A. sylvaticus*.

**Conclusion:**

Persistent enhanced lysozyme levels may prevent re- infection with trypanosomes.

## Introduction

Lysozyme (mucopeptide N- acetylmuramylhydrolase) is an enzyme lytic for the cell walls of certain bacteria, although this may not be an exclusive function (1). It is a protein with low molecular weight (15000), stable at acid pH and labile alkaline pH (2), and present in body fluids, cells and tissue of many living organisms where it appears to have a digestive and/ or defense function. Its mechanism of proteolysis has been described earlier (3, 4). It occurs in many fish tissues (5, 6), in rabbit spleen (7), snails (8), chicken lung (9) and polymorphonuclear leukocytes (10). In mammals, the blood granulocytes constituted the richest source of lysozyme (11). Cheng et al. (12) demonstrated haemolymph lysozyme activity in the snail, *Biomphalaria glabrata* and found that the enzyme was released from the phagocytes into the serum as a result of challenge by *Bacillus magaturium*. Powding and Davidson (13) studied lysozyme in the haemolymph of *Galleria mellonella* and *Bombyx mori*. Elevation in haemolymph lysozyme activity in *G. mellanella* larvae and other insects following injection of various materials represented the major part of the humoral defense mechanism against microbial invaders (14). The relationship between lysozyme and immunoglobulins as mediators of macrophage and plasma cell function is discussed (15). There is no doubt that the enzyme is of considerable importance in the immune defense system, being capable, in combination with complement and antibodies, of destroying pathogenic bacteria (16).

In this study the concentration of lysozyme in blood plasma of *Microtus agrestis, Clethrinomys glareolus*, *Apodemus sylvaticus*, BK rats and outbred white mice before and after infection with culture forms of *Trypanosoma microti, T, evotomys, T. grosi, T. lewisi and T. musculi* respectively were measured.

## Materials and Methods

Rodents, including *Microtus agrestis*, *Clethrinomy-s glareolus, Apodemus sylvaticus*, BK rats and outbred white mice were laboratory- bred in Salford University in England and maintained in cages on standard diets. Parasites, *Trypanosoma (Herpetosoma) microti, T, evotomys, T. lewisi and T. musculi* were maintained in Schneider′s *Drosophila* medium and *T. grosi* was cultured in graces medium (17).

Five animals of each species were bled from the tail under the Laboratory Animal License by collecting approximately 30 µl of blood into heparinised capillary tubes immediately and 3, 6, 12, 24, 48, 96 and more than 400 days after intraperitoneal inoculation with 5×105^5^ of their homologous trypanosome parasites, of which more than half were metacyclic trypomastigotes, in 0.2 ml of culture medium. Each capillary was sealed and centrifuged at 4700 g for 3 min to separate cells from plasma. The capillaries were broken just above the packed cells and the portion containing plasma was stored at – 20 °C until used.

Plasma lysozyme activities were measured by the agar plate method of Osserman and lawler (18). One gram of purified agar (Difco) was dissolved in 100 ml of 0.07 M phosphate buffer pH 6.9 (17 ml of 0.2 M Na2Hpo4; 965 ml distilled water) on hot plate stirrer. Fifty mg of *Micrococcus lysodeikticus* (Sigma) was suspended in the agar. Fifteen ml of the mixture were poured into each of six 9 cm Petri dishes, allowed to set and incubated overnight at 4 °C. Eight holes (4 mm in diameter) were punched in the agar in each dish and 15 µl of plasma sample dispensed into each well. The Petri dishes were then incubated at 37 °C for 3 h and overnight at 4 °C. The Petri dishes were then rinsed with PBS pH 7.2 and covered for 2 min with 1.5% tannic acid. The diameters of the transparent hydrolysis zones were then measured to the nearest 0.5 mm. The concentration of plasma lysozyme were calculated using a calibration curve constructed using dilutions of chicken egg- white lysozyme (Sigma) ranging from 1.9 to 2000 µg/ml.

The research was approved by the Ethical Committee of Salford University.

## Results

**Fig. 1:**
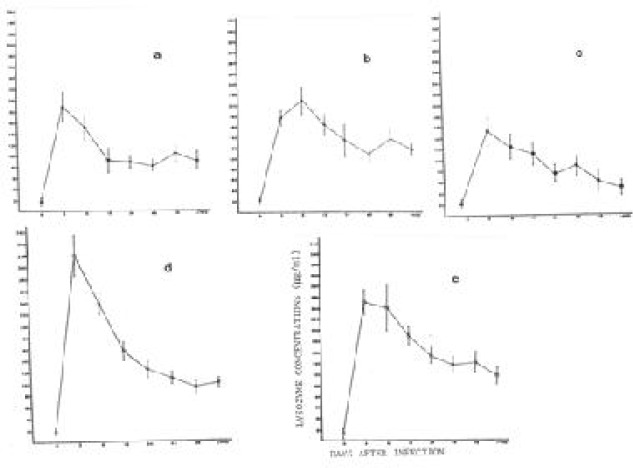
Lysozyme concentrations in the plasma of *M. agrestis* (a), *C. glareoulus* (b), *A. sylvaticus* ©, BK rats (d), and outbred mice (before infection B), 3, 6, 12, 24, 48, 96, and more than 400 days after infection

Concentration of lysozyme in the plasma of *Microtus agrestis, Clethrinomys glareolus, Apodemus sylvaticus*, BK rats and outbred white mice before and after inoculation with their homologous trypanosomes are shown in Table 1 and Fig. 1.

**Table 1:** Plasma lysozyme levels in *M. agrestis, C. glareolus, A. sylvaticus*, BK rats and Outbred mice (5animals per group) before and after inoculation with their homologous trypanosome parasites

Rodents	B I *	3**	6**	12**	24**	48**	96**	>400 DAYS**
***M. agrestis* With *T. microti***	17.1±7.58 (7.1-25.8)***	189.4±66.28 (119.4-271.9)	151.2±60.32 (100.0-241)	90.3±60.92 (32.75-160.2)	88.8±21.17 (66.3-119.4)	80.9±19.03 (74.6-100.0)	104.4±30.23 (83.9-151.0)	89.2±39.72 (74.6-134.2)
***C. glareolus* With *T. evotomys***	22.2±6.63 (14.3-30.8)	179.6±41.42 (134.2-241.7)	211.92±65.83 (160.2-324.3)	164.1±51.31 (119.4-241.7)	132.9±80.93 (74.6-271.9)	119.0±45.13 (55.6-180.1)	137.0±55.02 (66.3-180.1)	116.3±38.36 (88.9-160.2)
***A. sylvaticus* With *T. grosi***	20.6±6.62 (12.7-29.1)	152.9±74.38 (100.0-271.9)	123.4±52.56 (83.9-214.9)	111.0±46.78 (74.6-180.1)	73.4±33.45 (45.6-180.3)	89.2±39.43 (55.6-100)	52.9±13.13 (41.4-74.6)	48.4±10.30 (41.4-66.3)
**BK rats With *T. lewisi***	18.0±3.88 (17.1-23.0)	320.0±93.46 (214.9-435.2)	239.0±43.50 (180.1-288.3)	154.8±27.32 (119.4-180.1)	123.6±22.07 (100.0-150.2)	108.9±8.61 (100.0-119.5)	92.7±11.20 (74.6-100.0)	98.9±4.91 (94.3-100.0)
**Outbred mice With *T. musculi***	12.0±3.53 (7.98-17.15)	251.9±52.45 (180.1-324.3)	240.6±106.57 (105.1-386.9)	118.6±30.79 (161.1-241.7)	152.1±19.43 (134.2-180.1)	137.0±18.80 (119.4-180.1)	142.7±36.1 (100.0-180.1)	117.0±19.74 (100.0-134.2)

Mean followed by standard deviation and ranges (µg/ml)

* Before inoculation

** After inoculation

*** Figures in prentices are minimum and maximum levels of lysozyme in animals of each group

The level of lysozyme increased after inoculation of trypanosomes to their specific rodents and after 12 days reduced gradually, but stayed in the higher level than before inoculation. Hydrolysis zones of lysozyme in standard solutions and in plasma of rodents before and after inoculation are shown in Fig. 2a, b and c.

**Fig. 2a:**
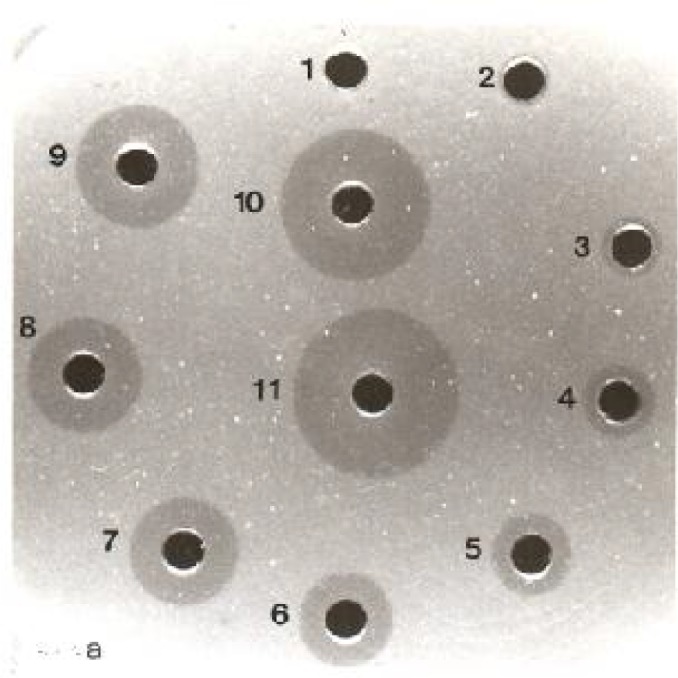
Hydrolysis zones of different concentrations of standard solutions: 1- 1.9, 2- 3.9, 3- 7.8, 4- 15.6, 5- 31.2, 6, 62.5, 7- 125, 8- 250, 9- 500, 10- 1000, 11, 2000 μg/ml

**Fig. 2b:**
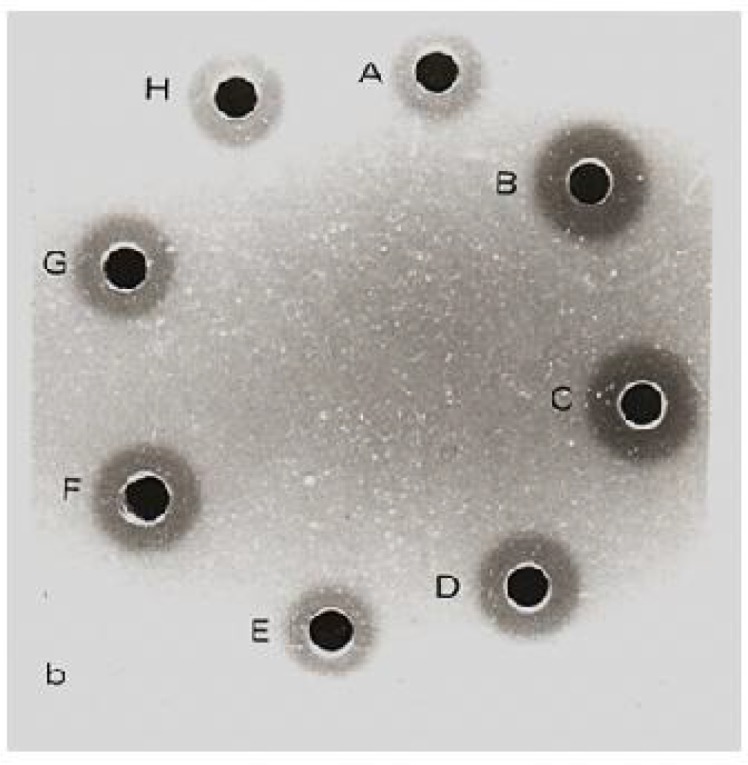
Hydrolysis zones of *A. sylvaticus* plasma: A, before inoculation B- 3, C- 6, D- 12, E- 24, F- 48, G- 96 and H, more than 400 days after inoculation with *T. grosi*

**Fig. 2c:**
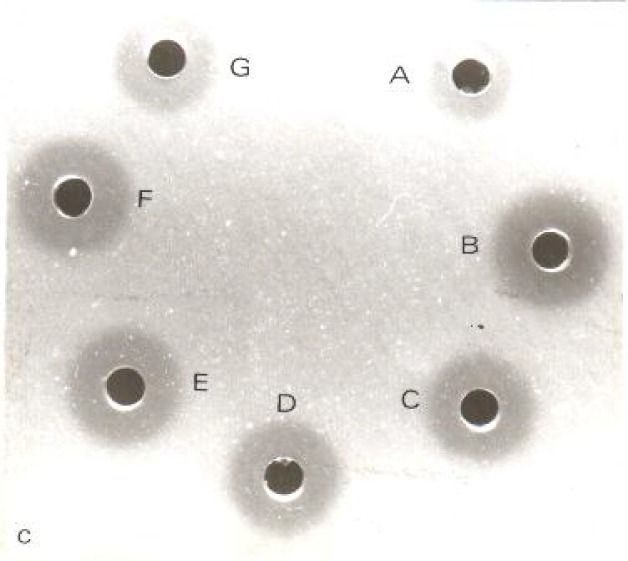
Hydrolysis zones of outbreed mice plasma: A before inoculation, B- 3, C- 6, D- 12, E- 24, F- 48, G- 96 days after inoculation with *T. musculi*

## Discussion

In mammals, lysozyme has been shown to be synthesized in and secreted into the blood by mononuclear phagocytes, particularly macrophages (19) and following antigenic stimulation of the immune system, the level of serum lysozyme increases significantly, for example in rabbits infected with *Trichinella spiralis* (20) and in the serum and urine of a dog with acute myeloid leukemia (21). Ingram and Molyneux (22, 23) reported a similar response in lizard with two to five fold increases in serum lysozyme of animals experimentally infected with *Leishman*ia. Daily lysozyme injection beginning on day 6 of *T. lewisi* infection in rats significantly reduced the number of circulating trypanosomes and this effect was dose dependent (24). Although these workers demonstrated that lysozyme did not cause lysis or immobilization alone or in combination with fibrinogen or rat serum, Usro and Ilard (25) demonstrated that *T. brucei* was quickly immobilized when exposed to lysozyme in vitro.

In the present study levels of lysozyme in the control plasma taken pre- injection were more than those reported for human sera (26). Upon trypanosome infection a ten to twenty fold increase in plasma lysozyme concentration occurred after 3- 6 days. The maximum value was found in rats infected with *T. lewisi* and the lowest in *A. sylvaticus* infected with *T. grosi*. Following the peak in activity during the first week of infection lysozyme levels fell but remained 2 to 10 times higher than control values for at least one year when the highest level was in mice, infected with *T. grosi*. Plasma lysozyme levels were thus well high after the rodents had their parasitaemias (7 to 13 days after infection in *Microtus, Clethrionomys* and *Apodemus*, BK rats 6- 12 weeks and in mice 3- 4 weeks.

## Conclusion

Lysozyme probably plays an important role in protecting rodents from re- infection.
